# *Veterinary Research* reviewer acknowledgement 2014

**DOI:** 10.1186/s13567-015-0222-z

**Published:** 2015-08-26

**Authors:** Michel Brémont, Élodie Coulamy

**Affiliations:** Veterinary Research, Unité de Virologie et Immunologie Moléculaires, INRA, Domaine de Vilvert, 78352 Jouy-en-Josas, cedex, France

## Abstract

The *Veterinary Research* editorial team would sincerely like to thank all of our reviewers who contributed to peer review for the journal in 2014.

**E.M. Abdelwhab**

Germany

**Mathias Ackermann**

Switzerland

**Alexandra Adams**

United Kingdom

**Alvaro Aguilar Setién**

Mexico

**Henri Agut**

France

**Raul Almeida**

United States of America

**Sonia Almeria**

Spain

**Irene Alvarez**

Argentina

**Mathieu Andraud**

France

**Elisavet Angelidou**

Greece

**Virginia Aragon**

Spain

**Clarice Arns**

Brazil

**Shawn Babiuk**

Canada

**Douwe Bakker**

Netherlands

**Mitchell Balish**

United States of America

**Keith Ballingall**

United Kingdom

**Herman Barkema**

Canada

**Michael Baron**

United Kingdom

**Walter Basso**

Switzerland

**Max Bastian**

Germany

**Andrew Bean**

Australia

**Douglas Begg**

Australia

**Graham John Belsham**

Denmark

**Ana Bento**

United Kingdom

**Are Berentsen**

United States of America

**Luiz Bermudez**

United States of America

**Jean**-**Francois Bernardet**

France

**Kateri Bertran**

United States of America

**Stephane Biacchesi**

France

**Stephen Bishop**

United Kingdom

**Barbara Blacklaws**

United Kingdom

**Damer Blake**

United Kingdom

**Sandra Blome**

Germany

**Rosa Bolea**

Spain

**Manuel Vitor Borca**

United States of America

**Thomas Boren**

Sweden

**Alex Bossers**

Netherlands

**Annemarie Bouma**

Netherlands

**Andrew Bowman**

United States of America

**Kevin Bown**

United Kingdom

**Filip Boyen**

Belgium

**Paulo Brandão**

Brazil

**Samantha Brandler**

France

**Volker Brinkmann**

Germany

**Jennifer Brisbin**

Canada

**Barbara Brito**

United States of America

**Collette Britton**

United Kingdom

**Erin Bromage**

United States of America

**Glenn Browning**

Australia

**Raymond Bujdoso**

United Kingdom

**Christian Burvenich**

Belgium

**Patrick Butaye**

Belgium

**Luis Calvinho**

Argentina

**Franco Cardone**

Italy

**Alexandre Caron**

Zimbabwe

**Chanhee Chae**

Korea, South

**Christophe Chevalier**

France

**Francesca Chianini**

United Kingdom

**Koen Chiers**

Belgium

**Rémi Choquet**

France

**Shafiqul Chowdhury**

United States of America

**Christine Citti**

France

**Axel Cloeckaert**

France

**Bertrand Collet**

United Kingdom

**Ellen Collisson**

United States of America

**Emmanuel Comoy**

France

**Andrew Conlan**

United Kingdom

**Timothy Connelley**

United Kingdom

**Franz Josef Conraths**

Germany

**Richard Cook**

United States of America

**Brian Cooke**

Australia

**Kevin Coombs**

Canada

**Eric Cox**

Belgium

**C. P. Binesh**

India

**Helen Crooke**

United Kingdom

**Sarah Culloty**

Ireland

**Cristina Cunha**

United States of America

**Adam Cunningham**

United Kingdom

**Charles Czuprynski**

United States of America

**Rkia Dardari**

Canada

**Stuart Dashper**

Australia

**Todd Davis**

United States of America

**Stephen Davis**

Australia

**Andrew Davison**

United Kingdom

**Michael Day**

United States of America

**Jeroen De Buck**

Canada

**Henri De Greve**

Belgium

**Sarne De Vliegher**

Belgium

**Sjaak de Wit**

Netherlands

**Lieven De Zutter**

Belgium

**Annemie Decostere**

Belgium

**Bernard Delmas**

France

**Piet Deprez**

Belgium

**Bert Devrient**

Belgium

**Jeroen Dewulf**

Belgium

**Adama Diallo**

Austria

**Isabelle Dimier**-**Poisson**

France

**Steven Djordjevic**

Australia

**Javier Dominguez**

Spain

**Charles Martin Dozois**

Canada

**Richard Ducatelle**

Belgium

**Mariette Ducatez**

France

**Christian Ducrot**

France

**Paul Duff**

United Kingdom

**Peter David Eckersall**

United Kingdom

**Torsten Eckstein**

United States of America

**Venessa Eeckhaut**

Belgium

**Susanne Eisenberg**

Netherlands

**Gary Entrican**

United Kingdom

**Ronald Erskine**

United States of America

**Nicolas Eterradossi**

France

**Øystein Evensen**

Norway

**James Evermann**

United States of America

**Claes Fellström**

Sweden

**Giancarlo Ferrari**

Italy

**Richard Ferrero**

Australia

**Thomas Ficht**

United States of America

**Bram Flahou**

Belgium

**Eduardo Flores**

Brazil

**Laurence Flori**

France

**Anthony Fooks**

United Kingdom

**Andrew Forbes**

United Kingdom

**Guillaume Fournié**

United Kingdom

**Caroline Frey**

Switzerland

**Joachim Frey**

Switzerland

**Sarah Gabriël**

Belgium

**Nellie Gagne**

Canada

**Barbara Gallagher**

Ireland

**Jean**-**Pierre Gangneux**

France

**Juan J. Garrido**

Spain

**Joseba M. Garrido**

Spain

**M Carolyn Gates**

United Kingdom

**Pierre Germon**

France

**Wilhelm Gerner**

Austria

**Laura Gillim**-**Ross**

United States of America

**Luis Gabriel Gimenez Lirola**

United States of America

**Elisabetta Giuffra**

France

**Liz Glass**

United Kingdom

**Anja Globig**

Germany

**Jose L Gonzales**

United Kingdom

**Marcelo Gottschalk**

Canada

**Kaare Græsbøll**

Denmark

**Simon Graham**

United Kingdom

**Philip Griebel**

Canada

**Martin H Groschup**

Germany

**Guiquan Guan**

China

**Luca Guardabassi**

Denmark

**Ronald Guenther**

Germany

**Carlton Gyles**

Canada

**Freddy Haesebrouck**

Belgium

**Kim Halpin**

Australia

**John Hammond**

United Kingdom

**David Hampson**

Australia

**Josee Harel**

Canada

**Balazs Harrach**

Hungary

**Christopher Heaney**

United States of America

**Andrew Hemphill**

Switzerland

**Uri Hershberg**

United States of America

**Marc Heyndrickx**

Belgium

**Frederic Hoerr**

United States of America

**Ole Højberg**

Denmark

**Jason Holland**

United Kingdom

**Martin Holland**

United Kingdom

**Jayne Hope**

United Kingdom

**Sawako Hori**-**Oshima**

Mexico

**David Horohov**

United States of America

**Emily Hotchkiss**

United Kingdom

**Jos Houdijk**

United Kingdom

**Valerie Hughes**

United Kingdom

**Munir Iqbal**

United Kingdom

**Naoki Isobe**

Japan

**Mario Jacques**

Canada

**Hugh Jones**

Australia

**Michael Jones**

United Kingdom

**Jorunn B Jørgensen**

Norway

**Gregers Jungersen**

Denmark

**Ramon A. Juste**

Spain

**Pete Kaiser**

United Kingdom

**Bernd Kaspers**

Germany

**Guenther Keil**

Germany

**Clayton Kelling**

United States of America

**David Kelton**

Canada

**Alyson Kelvin**

Canada

**Kathryn Kemper**

Australia

**Michael Kent**

United States of America

**David Kerr**

United States of America

**Anthony Keyburn**

Australia

**Hiroshi Kida**

Japan

**Donald King**

United Kingdom

**Peter Kirkland**

Australia

**Eyal Klement**

Israel

**Don Klinkenberg**

Netherlands

**Wolfgang Koester**

Canada

**Michael Kogut**

United States of America

**Nicholas Komar**

United States of America

**Peter Krawczel**

United States of America

**Naveen Kumar**

India

**Sachin Kumar**

India

**Gael Kurath**

United States of America

**Elisabetta Lambertini**

United States of America

**Benedicte Lambrecht**

Belgium

**Lucie Lamontagne**

Canada

**Frederic Lantier**

France

**Neus Latorre**-**Margalef**

United States of America

**Victoria Lawson**

Australia

**Ghislaine Le Gall**-**Recule**

France

**Ronan Le Goffic**

France

**Yves Le Loir**

France

**Sophie Le Poder**

France

**François Lefèvre**

France

**Alexandre Leitão**

Portugal

**Caroline Leroux**

France

**Martin Lessard**

Canada

**Randall Levings**

United States of America

**Zejun Li**

China

**Geneviève Libeau**

France

**Ann Lindberg**

Sweden

**John Lippolis**

United States of America

**Yoram Louzoun**

Israel

**Shuhong Luo**

China

**Blanca Lupiani**

United States of America

**Wenjun Ma**

United States of America

**Heba Mabrouk**-**Mostafa**

United States of America

**Rosangela Zacarias Machado**

Brazil

**N. James Maclachlan**

United States of America

**Vanesa Madan**

Germany

**Lone Madsen**

Denmark

**Dominiek Maes**

Belgium

**David Maggs**

United States of America

**Kathy Magor**

Canada

**Helena Maier**

United Kingdom

**Viviana Malirat**

Argentina

**Francois Malouin**

Canada

**Daniel Marc**

France

**An Martel**

Belgium

**Paolo Martelli**

Italy

**Ann Martens**

Belgium

**Hugh Mason**

United States of America

**Enric Mateu**

Spain

**James McGettigan**

United States of America

**Tom McNeilly**

United Kingdom

**Douglas Scott Merrell**

United States of America

**Francois Meurens**

Canada

**Simon Milling**

United Kingdom

**F. Chris Minion**

United States of America

**Rebecca Mitchell**

United States of America

**Petra Moebius**

Germany

**Fawzi Mohamed**

United States of America

**Maria Montoya**

United Kingdom

**Igor Morozov**

United States of America

**Ivan Morrison**

United Kingdom

**Liam Morrison**

United Kingdom

**Claude P. Muller**

Luxembourg

**Thomas Mueller**

Germany

**Claudio Murgia**

United Kingdom

**Michael Murtaugh**

United States of America

**Venugopal Nair**

United Kingdom

**Hans Nauwynck**

Belgium

**Mirjam Nielen**

Netherlands

**John Noh**

United States of America

**Romolo Nonno**

Italy

**Alejandro Núñez**

United Kingdom

**Jose I. Núñez**

Spain

**Norio Ohashi**

Japan

**Gaetano Oliva**

Italy

**Marinda Oosthuizen**

South Africa

**Klaus Osterrieder**

United States of America

**Tibor Papp**

Hungary

**Mark Parcells**

United States of America

**Nola Parsons**

South Africa

**Frank Pasmans**

Belgium

**Angela Pearson**

Canada

**Niels Pedersen**

United States of America

**Ben Peeters**

Netherlands

**Andres Perez**

United States of America

**Denis Pierard**

Belgium

**Gina Pighetti**

United States of America

**Patrick Pithua**

United States of America

**Karren Plain**

Australia

**Brandon Plattner**

Canada

**Yves Portejoie**

France

**Jacob Post**

Netherlands

**Edoardo Pozio**

Italy

**Marian Price**-**Carter**

New Zealand

**Maureen Purcell**

United States of America

**Auriol Purdie**

Australia

**Pascal Rainard**

France

**Thomas Bruun Rasmussen**

Denmark

**Johanna Marina Jacoba Rebel**

Netherlands

**Patrick Redig**

United States of America

**Robert Reiner**

United States of America

**Bertus Rima**

United Kingdom

**Espen Rimstad**

Norway

**Julian Rood**

Australia

**Marty Roop**

United States of America

**Nicolas Rose**

France

**Mirko Rossi**

Finland

**Ronan Rouxel**

France

**Franco Maria Ruggeri**

Italy

**Till Rümenapf**

Germany

**Edward Rybicki**

South Africa

**Ivan Rychlik**

Czech Republic

**Andrew Rycroft**

United Kingdom

**Armin Saalmueller**

Austria

**Konrad Sachse**

Germany

**Xavier Saelens**

Belgium

**Siba Samal**

United States of America

**Leonhard Schnittger**

Argentina

**Manuela Schnyder**

Switzerland

**Ynte Schukken**

United States of America

**Walter Schulz**-**Schaeffer**

Germany

**Manon Schuppers**

Switzerland

**Chris Secombes**

United Kingdom

**Luigi Sedda**

United Kingdom

**Mariela Segura**

Canada

**Hans**-**Martin Seyfert**

Germany

**Awad A. Shehata**

Germany

**M.S. Shekhar**

India

**I. Martin Sheldon**

United Kingdom

**Yujiang Shi**

United States of America

**Brian Shiels**

United Kingdom

**Craig Shoemaker**

United States of America

**Marion M Simmons**

United Kingdom

**Marek Sinkora**

Czech Republic

**Anja Sipka**

United States of America

**Michael Skinner**

United Kingdom

**Annemieke Smet**

Belgium

**Adrian Smith**

United Kingdom

**Kenneth Söderhäll**

Sweden

**Daesub Song**

Korea, South

**Stephanie Sonnberg**

United States of America

**Maria Rathmann Sørensen**

Denmark

**Marina Spinu**

Romania

**Tomasz Stadejek**

Poland

**David Stallknecht**

United States of America

**Mieke Steensels**

Belgium

**Mark Stevens**

United Kingdom

**Norbert Stockhofe**-**Zurwieden**

Netherlands

**Anne K Storset**

Norway

**Tanja Strive**

Australia

**Artur Summerfield**

Switzerland

**Lotta**-**Riina Sundberg**

Finland

**Gerd Sutter**

Germany

**Nicholas Svitek**

Kenya

**Raymond Sweeney**

United States of America

**Michael Szostak**

Austria

**Geraldine Taylor**

United Kingdom

**Gergely Tekes**

Germany

**Eileen Thacker**

United States of America

**Etienne Thiry**

Belgium

**Kim D. Thompson**

United Kingdom

**Peter Thompson**

South Africa

**Tijs Tobias**

Netherlands

**Panagiotis Tourlomousis**

United Kingdom

**Bryan Troxell**

United States of America

**Vikram Vakharia**

United States of America

**Thierry van den Berg**

Belgium

**Wim van der Hoek**

Netherlands

**Sylvia van Drunen Littel-van den Hurk**

Canada

**Koenraad Van Hoorde**

Belgium

**Filip Van Immerseel**

Belgium

**Kristien Van Reeth**

Belgium

**Debby van Riel**

Netherlands

**Vicky van Santen**

United States of America

**Michael van Straten**

Israel

**Wannes Vanderhaeghen**

Belgium

**Alain Vanderplasschen**

Belgium

**Marian Varady**

Slovakia

**Eva Vareckova**

Slovakia

**Jorge Velasco**-**Hernandez**

Mexico

**Elin Verbrugghe**

Belgium

**Elisabeta Vergu**

France

**Leen Verhaert**

Belgium

**Mafalda Viana**

United Kingdom

**Stefan Vilcek**

Slovakia

**Vladimir Vrba**

Czech Republic

**Bettina Wagner**

United States of America

**Thomas Wahli**

Switzerland

**Cheryl Waldner**

Canada

**Bernard Wasinski**

Poland

**Pierre Wattiau**

Belgium

**Richard Webby**

United States of America

**Scott Wells**

United States of America

**C Jane Welsh**

United States of America

**Dirk Werling**

United Kingdom

**Stephen White**

United States of America

**Gary Whittaker**

United States of America

**Paul Wigley**

United Kingdom

**Rebecca Wilkes**

United States of America

**Robert Wolf**

Canada

**Yongjun Yu**

United States of America

**Mark Zabel**

United States of America

**Jianqiang Zhang**

United States of America

**Annetta Zintl**

Ireland

**Siamak Zohari**

Sweden

